# Effects of Intradermal Sterile Water Injections in Women with Low Back Pain in Labor: A Randomized, Controlled, Clinical Trial

**DOI:** 10.4274/balkanmedj.2016.0879

**Published:** 2018-03-15

**Authors:** Refika Genç Koyucu, Nurdan Demirci, Ayşe Ender Yumru, Süleyman Salman, Yavuz Tahsin Ayanoğlu, Yıldız Tosun, Cihangir Tayfur

**Affiliations:** 1 Department of Maternity and Gynecology Nursing, Beykent University School of Medicine, İstanbul, Turkey; 2 Department of Maternity and Gynecology Nursing, Marmara University School of Medicine, İstanbul, Turkey; 3 Department of Maternity and Gynecology, University of Healty Sciences, Şişli Etfal Training and Research Hospital, İstanbul, Turkey; 4 Department of Obstetric and Gynecology, University of Healty Sciences, Gaziosmanpaşa Taksim Training and Research Hospital, İstanbul, Turkey; 5 Esenyurt State Hospital, İstanbul, Turkey

**Keywords:** Analgesia, injections, labor, obstetrical, pain, randomized controlled trial

## Abstract

**Background:**

In addition to the pain caused byuterine contractions during labour, continuous and severe back pain is observed in 33% of women. Several pharmacological and nonpharmacological methods are available for managing this pain. Sterile water injection is considered as alternative method for nonpharmacological pain management.

**Aims:**

To assess the satisfaction level and effectiveness of sterile water injection for back pain among women in labour.

**Study Design:**

Randomized controlled trial.

**Methods:**

A total of 168 term, healthy women with labour pain and severe back pain were randomized into the sterile water injection (study) and dry injection (placebo) groups. Injections were applied to the rhombus of Michaelis in the sacral area. Pain scores were assessed at 10, 30, 60, 120, and 180 min using a visual analogue scale. Additionally, the need for epidural analgesia, Apgar score, mode of delivery, time of delivery, maternal satisfaction, and breastfeeding score were assessed.

**Results:**

The mean back pain scores at 30 min after injections were significantly lower in the study group (study group: 31.66±11.38; placebo: 75±18.26, p<0.01). The mean decrease in pain scores after 30 min according to baseline was significantly higher in the study group (study group: 54.82±7.81; placebo: 13.33±12.05, p<0.01). The need for epidural analgesia, time of delivery, mode of delivery, and Apgar and breastfeeding scores were similar in both groups. Maternal satisfaction from the analgesic effect was significantly higher in the study group (study group: 84.5%; placebo: 35.7%, p<0.01).

**Conclusion:**

The application of sterile water injection is effective for relieving back pain in the first stage of labour and has a sufficient satisfaction level among women.

Pain during labour is a physiological and subjective phenomenon. Women experience pain caused by diffused abdominal spasms, visceral pain, and uterine contractions during the first stages of labour. In the second stage of labour, women also feel a sharper and continuous somatic pain (1). There are considerable variations in the localization and degree of labour pain. In addition to the pain associated with contractions, 33% of women report experiencing severe discomfort in the lower back, which is most intense during contractions and often painful between contractions (2). Contrary to the rhythmic pain caused by uterine contractions, back pain during labour is caused by distension of neighbouring visceral and neural structures and felt throughout labour (2).

Various pharmacological and nonpharmacological methods are available for back pain during labour. The main emphasis of pharmacological methods is the elimination of the physical sensation of labour pain, whereas the emphasis of nonpharmacological methods is largely on preventing suffering (3).

Alternative analgesic applications are needed in labour pain management in Turkey because of high rates of cesarean sections (C/S), potential side effects, and limited accessibility of regional analgesia methods. Sterile water injection (SWI) is an alternative nonpharmacological method used to treat severe visceral organ pain (such as chronic myofascial pain, urinary colic, and labour pain) (4,5,6). SWI has increased in popularity for labour pain treatment, especially in the last two decades. This method does not involve the use of medications. Also, it does not restrict maternal mobilisation. Additionally, it is easy to administer and is cost-effective. Therefore, this novel clinical study aimed to assess the satisfaction level and effectiveness of SWI for back pain among women in labour.

## MATERIALS AND METHODS

This randomised prospective study was performed between June 2013 and March 2014 in the maternity clinic. Written approval for the study was obtained from the ethics committee of the hospital (9 July 2014; #63). The study included a placebo-treated patient group (control group) with dry injections and an SWI group (study group). This study was registered at ClinicalTrials.gov (#NCT002697994). The study criteria are shown in Table 1.

**Figure 1:**
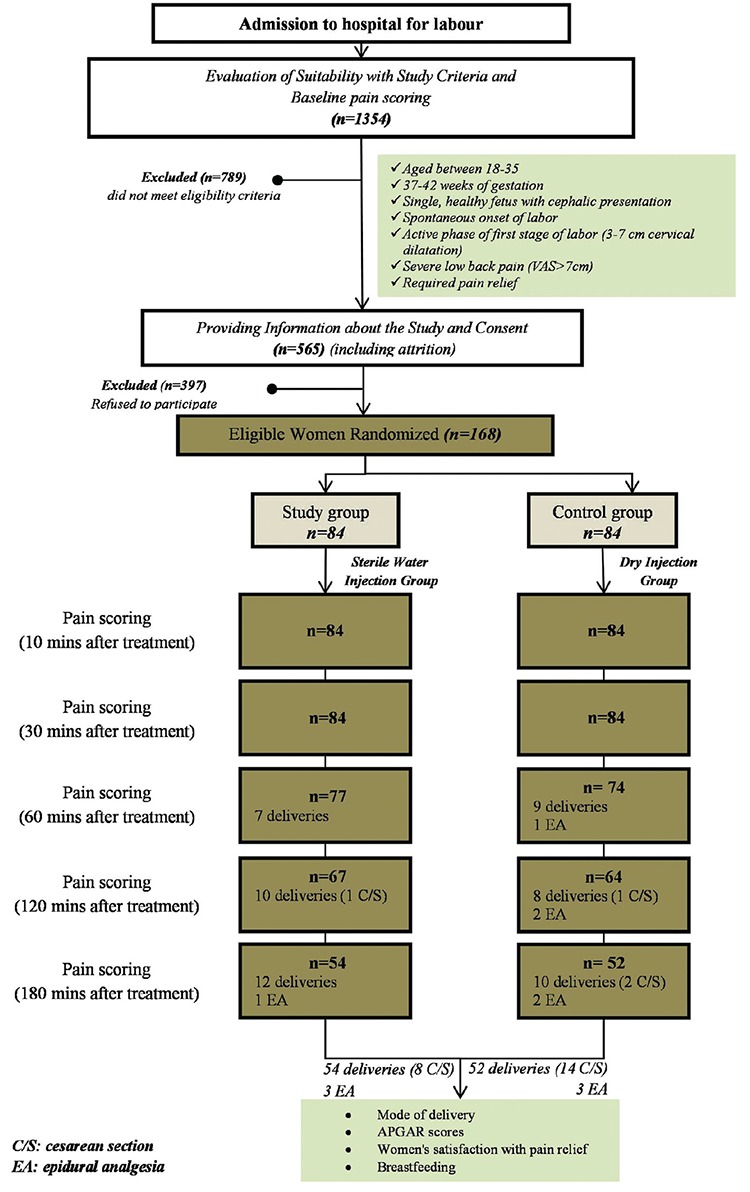
Randomization and participant flow. Stages of the study from the time from admission of cases till the end of the study (evaluation of cases, information about the study, receiving consents for the study, randomization, changes in the number of cases during this process,cesarean section rates,and needs for epidural analgesia), criteria for the study, and aims of the study are summarized.

Participants were randomly assigned to the study group (four intradermal injections of 0.1 mL of sterile water into the skin surrounding the rhombus of Michaelis over the sacral area). The rhombus of Michaelis is a diamond-shaped area, over the posterior aspect of the pelvis, formed by the dimples of the posterior superior spines of the ilia, the lines formed by the gluteal muscles, and the groove at the distal end of the vertebral column. The first injections were given on the posterior superior iliac spines (both sides) and the second injections 1 cm medial and 1-2 cm inferior to the first injections (on both sides), using an insulin needle (Figure 1). Participants in the control group received four dry injections in the same region using an insulin needle. The study outcomes are shown in Table 2.

Statistical power analysis was used to calculate the required sample size. The sample size was estimated in accordance with an earlier, similar study. According to this study, the means and standard deviations of the pain scores for the sterile water and placebo groups were 52.3±23.6 and 69.7±23.4 respectively. Given a true difference in the pain score of -17.40 between the study and control groups and a statistical power of 0.8, to reject a null effect at the 0.05 significant level, it was calculated that at least 32 cases would be needed for each group.

**Figure 2:**
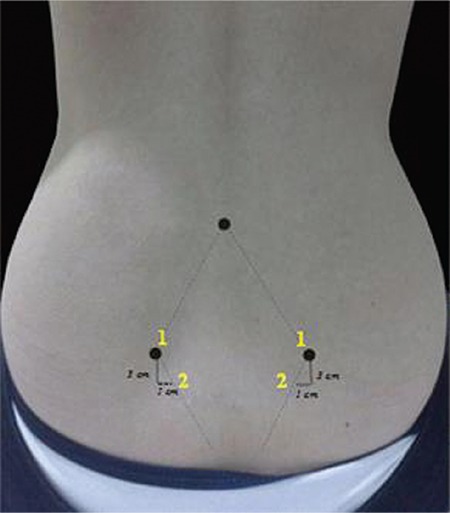
Localization and application of injections. It demonstrates the rhombus of Michaelis region and location of injections. The rhombus of Michaelis region is the region in the shape of an equilateral quadrangle, which is located among posterior superior iliac spines, gluteal muscles, and spinous process of vertebra L4; 3 cm lower and 1 cm medial from superior iliac spines and spines were marked. Injections were given to both groups simultaneously and at the peak point of contractions by two midwives.

Compliances to the research criteria and pain scores concerning back pains of women admitted with labour pain were determined (n=1354). Cases that did not comply with study criteria or that had a back pain score of <7 were excluded from the study (n=789). Cases that complied with the criteria were informed both verbally and in writing, and their consents were obtained. Cases that did not provide informed consent were also excluded (n=397). Compliant cases were randomised into a study group (sterile water, SWG, n=84) and a control group (dry injection, DIG, n=84). Randomization was done using a computer, and envelopes containing randomisation results were kept sealed in the delivery room. Block randomisation was used to equilibrate the groups (Figure 2).

Injections were given to both sides simultaneously and at the peak point of contractions by two midwives. The groups into which the women were placed was known only to the midwives. The researchers that delivered and undertook the pain scoring were not in the room at the time of the injections. Visual analogue scale (VAS) was used for pain scoring. Pain scores were evaluated before and 10, 30, 60, 120, and 180 min after injections. Pain scoring for each woman was performed by a single researcher. This researcher did not know the group to which the women had been assigned. A different scale, which had not been used earlier, was used for all evaluations of a case. Women who gave birth before completion of their pain scorings were not excluded from the study. Fifty-four cases in the study group and 54 cases in the control group were present for the 180 min pain score. Pain scores at different scoring times were evaluated among cases that had not yet given birth (Figure 2). Cases that needed additional analgesia during labour were given epidural analgesia, and further pain scorings of these cases were not performed. The rate and need for epidural analgesia in the groups were recorded. Follow-up labour was performed by midwives and obstetricians, and knowledge concerning the labour was recorded in the partograph recommended by the Ministry of Health. Delivery was done by the midwives and obstetricians. The mode of delivery was recorded, and the rate of C/S of groups was determined. A satisfaction questionnaire concerning the pain-relieving method was completed by the women following the delivery. Additionally, the success of breastfeeding was evaluated during the first hour and within the first day of the postpartum period using the Infant Breastfeeding Assesment Tool (IBFAT). Data collection tools and documented data are shown in Table 3.

To assess the adequacy of randomization, demographic and other baseline characteristics were analyzed for comparison between groups. Differences in the VAS pain score between the two groups were analyzed using the Student t-test. The Mann-Whitney U test was used for non-parametric data and the chi-squared test for categorical variables.

## RESULTS

A total of 1354 consecutive women presenting in labour were screened for participation in this study. Of these, 789 women did not meet the study criteria and were excluded, 565 women consented, and 397 women declined study participation. Thus, 168 women were randomized to the study (n=84, 4×0.1 mL of sterile water) and control groups (n=84, 4×0.1 mL of dry injection). Both groups were similar in terms of age, parity, gestational age, body mass index, and other clinical data (Table 4).

**Figure 3:**
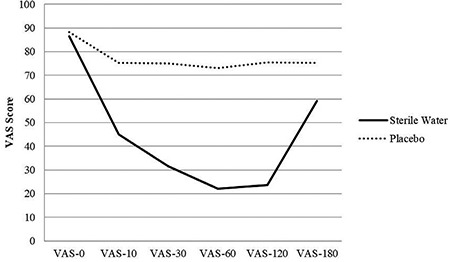
Mean pain scores and pain score reduction between the two groups. Alterations in mean pain scores (0, 10, 30, 60, 120, and 180 min) after injections are demonstrated.

Table 5 and Figure 3 show the mean pain scores during labour as well as changes in the mean pain scores. Pain scores at 30 min after injections, which was the primary outcome of the study, were significantly lower in the study group than the control group (SWG: 31.66±11.38; DIG: 75±18.26, p<0.01). The mean decrease in pain scores after 30 min according to baseline was significantly higher in the study group than the control group (SWG: 54.82±7.81; DIG: 13.33±12.05).

Some women were excluded from the 60, 120, and 180 min pain scoring because they gave birth before the completion of the study or due to a need for epidural analgesia. The mean pain scores at 10, 60, 120, and 180 min after injections were significantly lower in the study group than the control group. The decrease in mean pain scores was significantly greater in the study group than the control group (Table 5 and Figure 3). 

Table 6 shows the need for epidural analgesia, modes of delivery, and neonatal Apgar scores of the cases. Although it was statistically insignificant, the rate of C/S was approximately two-fold higher in the control group compared with the study group (SWG 10.7%; DIG: 20.2%, p=0.08). The need for epidural analgesia was similar between the two groups (SWG: 4.76%; DIG: 9.52%, p=0.231). Although it was statistically insignificant, the time from the first injection till the labour was shorter in the study group than the control group (SWG: 170±53.4 min; DIG: 180±62.65 min, p=0.06). Neonatal Apgar scores were similar in both groups. 

The results concerning satisfaction from analgesic effect and lactation are given in Table 7. The satisfaction levels from the analgesic injection, wish for reuse in further pregnancies, and thoughts of recommending it to others were significantly higher in the study group than the control group (p=0.01). The IBFAT scores at postpartum first hour and first day were similar in both groups.

## DISCUSSION

Labor pain is one of the most significant pains experienced by women and concerns about pain during labour remains a hot topic. Various noninvasive (hydrotherapy, acupuncture, yoga, music, counterpressure, acupressure, relaxation, breathing techniques, positioning/movement, and transcutaneous electrical nerve stimulation) and pharmacologic treatments (nitrous oxide, opioids, and regional analgesia techniques: spinal, epidural, and combined epidural analgesia) are available for managing labour pain. Regional anaesthesia methods (epidural, spinal, or epidural-spinal combination) are considered the most popular and most effective methods for addressing labour pain (7,8). Although regional anaesthesia methods provide potent analgesia, they have disadvantages, such as causing immobilisation of the mother and a prolonged and instrumental labour (9). These methods are also less accessible in developing countries and those with an extensive rural population, such as Turkey. The search for an analgesic method that is effective, cost-effective, and easy to apply and access is ongoing. SWI has become increasingly popular for these reasons. The number of studies demonstrating positive results in the application of this method has increased. In the majority of these studies, SWI and placebo (isotonic, dry injection, standard care) were compared, as in the present study (10,11,12,13,14,15). In some studies, other alternative treatment methods, such as acupuncture and transcutaneous electrical nerve stimulation, were compared with SWI (16,17). In these studies and the present study, the analgesic effectiveness of sterile water was assessed mostly by VAS at multiple time intervals (10-180 min). It was determined that SWI provided a significant decrease in pain according to the basal pain level. Additionally, SWI has been demonstrated to be effective for labour pain in two meta-analyses (18,19). In the present study, a significant decrease in the pain score was observed at 10 min after SWI; this decrease was by greater than 50% at the 30, 60, and 120 min timepoints. Despite a decrease in its magnitude, the pain-reducing effect of SWI continued at the 180 min. The results of the present study seem to be consistent with the findings of other studies, and SWI was found to be effective for relieving back pain during labour.

Previous studies have reported that the analgesic effectiveness of SWI lasted for approximately 2 h, and can be repeated if necessary (14,15,16,18,19). The analgesic effectiveness of SWI was found to be highest between 30 and 120 min. 

Novel methods used to treat labour pain bring anxieties about their possible effects on labour, mode of delivery, and the fetus/newborn. There has been an increase in C/S rates worldwide, and efforts are being made to reduce this trend. C/S rates are currently above 15% in 69 (50.4%) countries. Although this rate differs greatly among different regions in Turkey, it reached 50.4% in 2013, up from 21% in 2001 (20). Analgesic methods used in labour pain should not cause an increase in C/S rates. Previous studies that evaluated SWI in labour pain reported that SWI application did not affect the mode of delivery (17,18,20). Additionally, SWI has been found to decrease the C/S rate in a systematic review of eight Randomized Control Studies. In the present study, although the C/S rates were lower in the case of SWI, this did not reach statistical significance (SWI: 10.7%; placebo: 20.1%, p=0.088). Lee et al. (21) initiated the study entitled “Impact on caesarean section rates following injections of sterile water (ICARIS)”, which was planned to last between 2-3 years with a hypothesis that the positive effects of SWI might decrease the C/S rates. Although recent studies have shown that regional analgesia does not affect the C/S rate, this issue continues to be debated. Additionally, these methods cause the prolongation of the second stage and instrumental labour (22). In the present study, the time from SWI until the birth was similar for the study and control groups, similar to the results of Wiruchpongsanon (15).

Neonatal Apgar scores in the study group were similar to those of placebo group. Moreover, it was observed that the sucking characteristics of newborns at postpartum first day were similar between the two groups. Consistent with previous studies, we found that SWI is an effective method of relieving the pain, without influencing the mode of delivery or duration of labour or having a negative effect on the newborn. Additionally, SWI had no detectable side effects on the mother, such as immobilisation, changes in vital signs, or unconsciousness.

Satisfaction from the analgesic effect, thought of recommending SWI to others, and wish to reuse it in future pregnancies were significantly higher in the study group than the control group (SWI satisfaction: 84.5%; control group satisfaction: 35.7%, p<0.01). 

In conclusion, SWI for labour pain is a simple, cost-effective, easily accessible, safe, and promising method in developing countries having higher rates of total and rural births and C/S. SWI seems to be an efficient and simple method for treating antagonising low back pain during labour, especially in low-resource settings. SWI provides an analgesic effect lasting up to 120 min. SWI does not affect the state of consciousness and can decrease the need for epidural analgesia. SWI does not limit maternal mobility. Moreover, SWI does not interfere with labour progress or the ability to push and can be done at home births and in out-of-hospital birth centres by a nurse or midwife, without the need for an anesthesiologist. Also, unlike narcotics, SWI does not lead to vomiting or neonatal depression, and it does not cause drowsiness and nausea, which nitrous oxide does. It is believed that more extensive studies are required in Turkey and other countries to demonstrate the effects of SWI on the mode of delivery and C/S rates. We are now considering a multicenter study addressing SWI during labour, to be undertaken in two state-run hospitals in Turkey.

## Figures and Tables

**Table 1:**
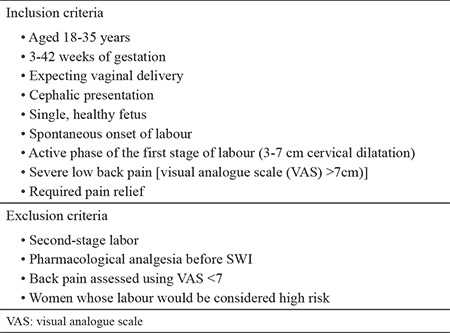
The criteria for the inclusion and exclusion of patients to the trial

**Table 2:**
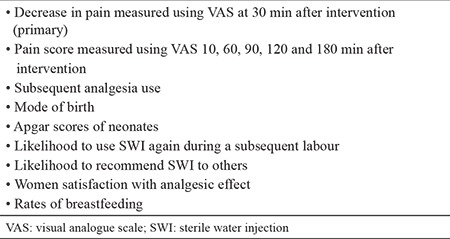
Study outcomes

**Table 3:**
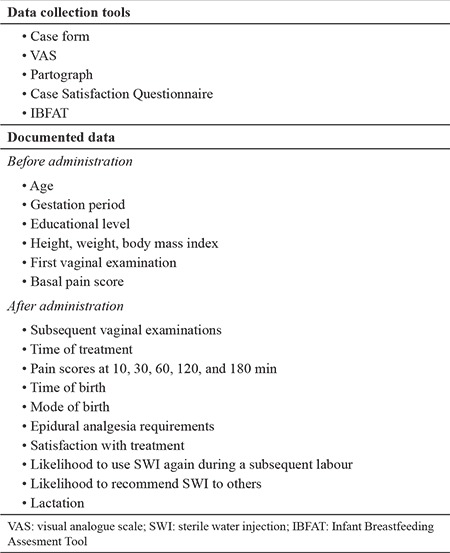
Data collection tools and documented data

**Table 4:**
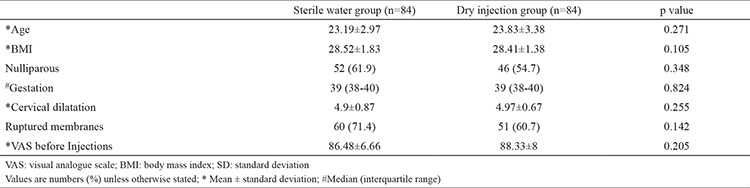
Baseline demographic and clinical characteristics for each group of participants

**Table 5:**
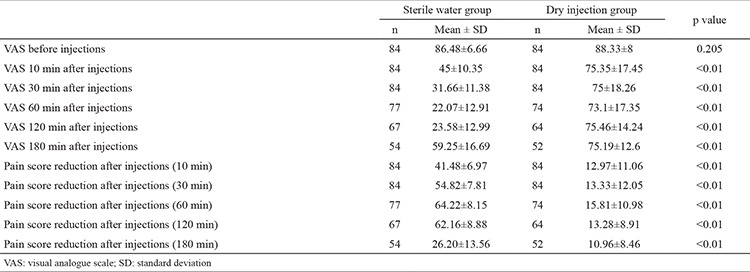
Mean pain scores and pain score reduction between the two groups

**Table 6:**
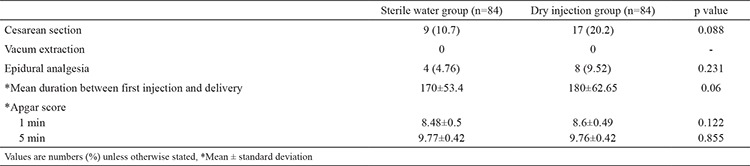
Need for epidural analgesia, mode of delivery, and Apgar scores

**Table 7:**
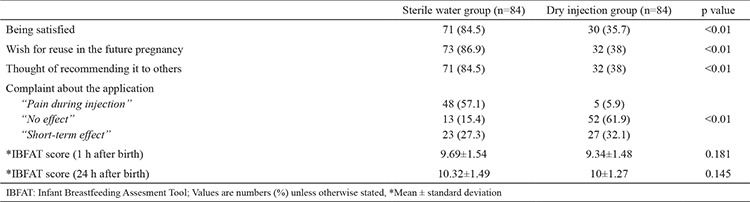
Maternal satisfaction and breastfeeding scores for the two groups
